# An Efficient Method for Identifying Gene Fusions by Targeted RNA Sequencing from Fresh Frozen and FFPE Samples

**DOI:** 10.1371/journal.pone.0128916

**Published:** 2015-07-01

**Authors:** Jonathan A. Scolnick, Michelle Dimon, I-Ching Wang, Stephanie C. Huelga, Douglas A. Amorese

**Affiliations:** NuGEN Technologies, Inc., San Carlos, California, United States of America; The University of Texas MD Anderson Cancer Center, UNITED STATES

## Abstract

Fusion genes are known to be key drivers of tumor growth in several types of cancer. Traditionally, detecting fusion genes has been a difficult task based on fluorescent *in situ* hybridization to detect chromosomal abnormalities. More recently, RNA sequencing has enabled an increased pace of fusion gene identification. However, RNA-Seq is inefficient for the identification of fusion genes due to the high number of sequencing reads needed to detect the small number of fusion transcripts present in cells of interest. Here we describe a method, Single Primer Enrichment Technology (SPET), for targeted RNA sequencing that is customizable to any target genes, is simple to use, and efficiently detects gene fusions. Using SPET to target 5701 exons of 401 known cancer fusion genes for sequencing, we were able to identify known and previously unreported gene fusions from both fresh-frozen and formalin-fixed paraffin-embedded (FFPE) tissue RNA in both normal tissue and cancer cells.

## Introduction

Cancer cells frequently contain chromosomal rearrangements that lead to the formation of fusion genes expressed in the cell [1]. These fusion genes can act as drivers for cell growth. For example, the Philadelphia Chromosome rearrangement, originally identified in chronic myelogenous leukemia [2] (CML), is the result of a translocation between chromosomes 9 and 22 which leads to the expression of a fusion gene combining the BCR and ABL kinases [3]. While the BCR-ABL fusion gene leads to uncontrolled cell growth, when the fusion gene is identified in a patient, CML can be treated successfully with tyrosine kinase inhibitors.

Gene fusions are typically identified in cells by fluorescent *in situ* hybridization (FISH), a technique in which selected regions of chromosomes are fluorescently labeled through the hybridization of specific oligonucleotide probes. Aclinician must then visually identify two chromosomal regions that have rearranged in a known pattern. FISH has many problems as a technique including being a difficult, low throughput procedure, required knowledge of both gene fusion partners, low spatial resolution, the availability of the necessary fluorescent probes and the need for highly trained personnel to decide if a fusion event has taken place [4]. These difficulties apply to both clinical and research laboratories, thus limiting the potential understanding of gene fusion events in human biology.

**Fig 1:**
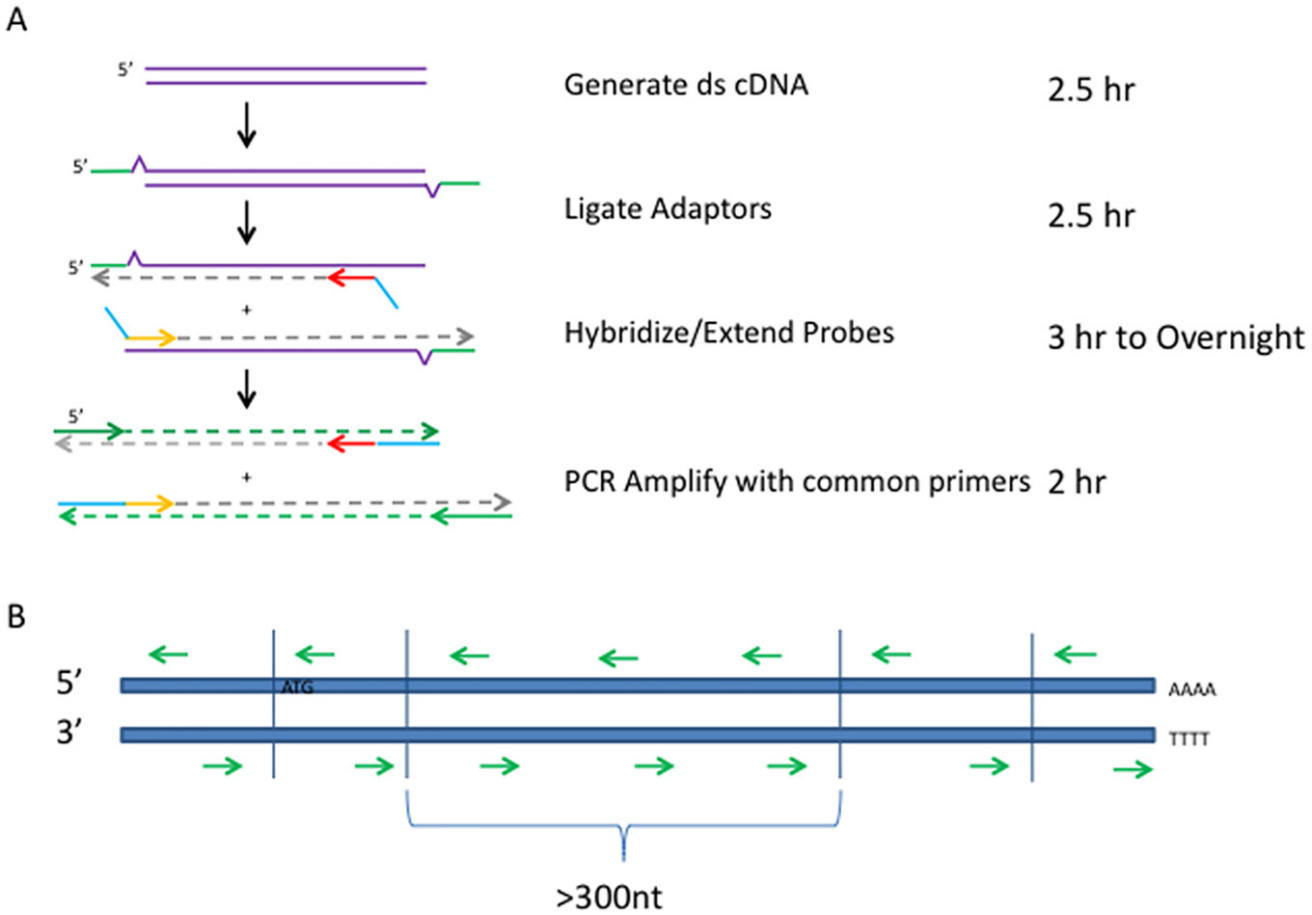
Description of the Ovation Target Enrichment System. (A) Experimental steps of the assay and time required for each step. Adaptors (green) are ligated on to generated double stranded cDNA (ds-cDNA). Probes (shown in red and yellow) are hybridized to target cDNA and extended with a polymerase (dashed grey lines). All probes have common tail sequences (blue), which are used as priming sites along with adaptor sequences in subsequent library amplification PCR steps. (B) Example of probe positioning across different exons in a full length double stranded cDNA. Each exon (demarked by blue vertical lines) will have probes (green arrows; arrow points in the 3’ direction) designed to hybridize near the predicted exon-exon junctions. Exons larger than 300 nucleotides (nt) may have additional probes tiled along the length of the exon to obtain more complete sequence coverage. Probes are designed against both strands of the cDNA to enable identification of gene fusions when only one of the pair of genes is targeted. Translation start sequence (ATG) and poly A tail are labeled.

One recent alternative to FISH when studying gene fusions is high throughput sequencing. In particular, RNA sequencing (RNA-Seq) has been used to identify gene fusions that are transcribed into RNAs within various cells [5]. Software has been specifically developed to identify gene fusion events in RNA-Seq data [6] and many previously unknown gene fusions have been identified this way. However, while RNA-Seq is a powerful tool for identifying gene fusion transcripts, it is currently cost prohibitive to sequence RNA from a tumor sample, in which only a small fraction of the total cells are expressing the gene fusion, and obtain enough sequencing reads to identify those fusion events. Here we describe an innovative new assay for gene fusion discovery and verification. Based on Single Primer Enrichment Technology (SPET, Fig 1A), we target particular RNAs for sequencing, thus reducing the number of uninformative sequencing reads and increasing the sensitivity of gene fusion detection compared to standard RNA-Seq methods. The SPET based assay is easy to use, has low RNA input requirements and can be used with RNA from formalin fixed, paraffin embedded (FFPE) tissue, which is important for clinically relevant samples. In addition, the assay is fully customizable to target any gene or set of genes in any genome. To show the value of this technology, we have created a panel of probes to target 401 human genes based on gene fusion events found in the Wellcome Trust databases [1], Chimerdb 2.0 [7] and The Cancer Genome Atlas (http://cancergenome.nih.gov/) (full list in S1 Table) and have used this probe panel to identify known and previously unidentified gene fusion events in cancer cells lines and human whole brain RNA.

## Results

### Targeted RNA-Sequencing

In order to test the ability of the Ovation Fusion Panel Target Enrichment System assay to effectively target RNAs of interest, we ran the assay using 100 ng of total RNA from the H2228, lung adenocarcinoma cell line to see what percentage of sequencing reads were derived from the targeted genes. The resulting library was sequenced on an Illumina MiSeq with paired end 75bp reads. 92.5% of reads mapped back to the genome with 96% of those reads mapping to the targeted RNAs. These results indicate a strong selection for targeted RNAs by the Ovation Fusion Panel Target Enrichment System. Comfortable that we were selecting almost exclusively for the sequences we were interested in, we next looked to identify gene fusions.

### Gene Fusion Identification in H2228 Cells

A key feature of our assay is the ability to identify gene fusions even when only one of the partners has been targeted. This is achieved by through the use of Single Primer Enrichment Technology (SPET), which only requires the hybridization of a single primer in order to obtain sequencing information from the downstream RNA. H2228 cells carry one allele of the *ALK* gene that is fused to *EML4* and the other allele is fused to *PTPN3*. In our targeting panel we have probes targeting *ALK* and *EML4*, but not *PTPN3* so this served as a good test for identifying fusion transcripts for which only one of the genes was directly targeted. The sequencing data were analyzed for the presence of gene fusions (see materials and methods) and we were able to identify both the predicted *ALK-EML4* and the *ALK-PTPN3* gene fusions with 512 and 20 reads respectively for each fusion out of a total of 4.5 million reads.

### Targeted RNA Sequencing Identifies More Fusions than Standard RNA-Seq

**Fig 2:**
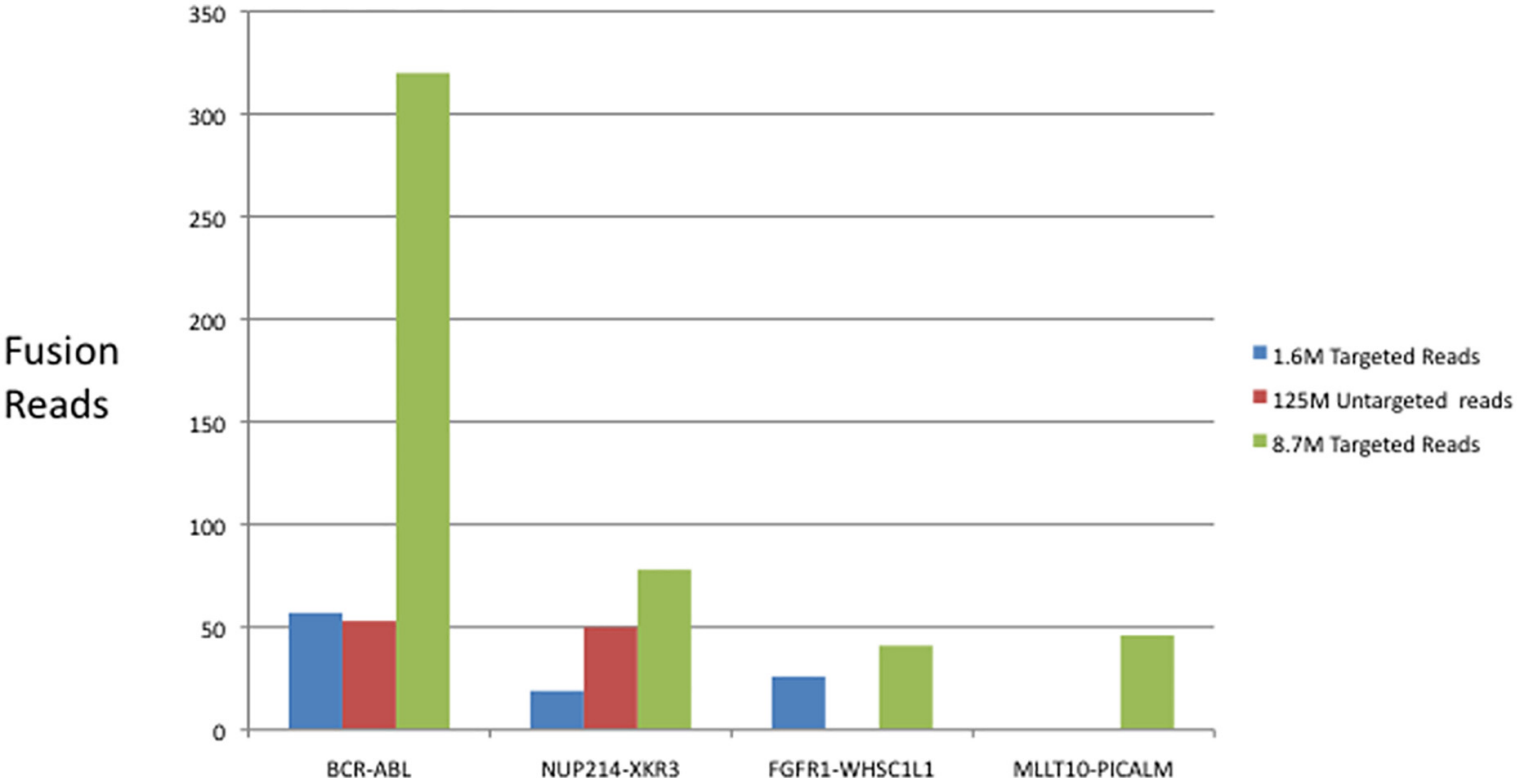
Ovation Fusion Panel Target Enrichment System identifies known and novel gene fusion events in Universal Human Reference RNA. The number of sequencing reads determined to be derived from gene fusions in two different targeted sequencing libraries (blue and green) compared to the events identified in a standard, untargeted RNA-Seq library. The untargeted library (red) consists of 125 million total sequencing reads while the targeted libraries consist of 1.6 and 8.7 million sequencing reads.

We next wanted to compare our results to those of standard RNA-Seq experiments to test how many sequencing reads were necessary to identify gene fusion events when using the Ovation Fusion Panel Target Enrichment System relative to a non-targeted RNA-Seq approach. Universal Human Reference RNA (UHR) contains RNA from ten different cancer cell lines. Many standard RNA-Seq experiments have been performed using UHR RNA and these experiments have identified a number of gene fusion events [8]. Our 401 gene panel contains probes that target two of the fusions previously shown to exist in UHR RNA, *BCR-ABL* and *NUP214-XKR3*. 100 ng of UHR total RNA was used to prepare sequencing libraries with Ovation Fusion Panel Target Enrichment System. Libraries were sequenced on either the Illumina MiSeq or HiSeq and analyzed with SOAPFuse [6]. Our initial tests utilizing 1.6M sequencing reads resulted in the identification of both *BCR-ABL* and *NUP214-XKR3* fusion transcripts. In addition, we also identified an *FGFR1*-*WHSC1L1* gene fusion, which has been reported as a recurring fusion in breast cancer [9], but has not been reported in UHR RNA. Deeper sequencing of another library to 8.7M reads on the Illumina HiSeq led to the further identification of another previously unreported UHR gene fusion, *MLLT10* fused to *PICALM*. Having identified a set of gene fusions in UHR RNA, we compared the number of fusion reads in these genes detected by SOAPFuse in our datasets to those of a publically available RNA-Seq dataset that consists of 125M paired end sequencing reads [10]. As seen in Fig 2, when comparing 1.6M Ovation Fusion Panel Target Enrichment System sequencing reads (blue bars) to 125M standard RNA-Seq sequencing reads (red bars), fusion read counts are comparable for the *BCR-ABL* fusion. The 1.6M targeted sequencing reads has fewer reads for *NUP214-XKR3*, which is expected because our targeting panel only contains probes for *NUP214* and not *XKR3*. The standard RNA-Seq does not contain any detectable reads for *FGFR1-WHSC1L1* nor any reads for *MLLT10-PICALM*, while the targeted sequencing approach described here identified both of these fusion events. Both of these previously undescribed fusions were verified by RT-PCR and Sanger sequencing. In summary, by using a targeted sequencing approach, we were able to identify known gene fusion events in UHR RNA while using only 1.3% of the sequencing reads necessary to identify approximately the same number of fusion reads in a standard RNA sequencing experiment. In addition we were able to identify previously undocumented gene fusion events using the targeted approach that were not identified in standard RNA sequencing, even though the targeted sequencing had 14–78X fewer sequencing reads. The inability of standard RNA sequencing to identify fusion transcripts in a cell line that has been studied in detail suggests that even samples for which deep RNA sequencing data already exists should be tested again specifically for the presence of fusion transcripts.

### Human Tissue Gene Fusions

**Fig 3:**
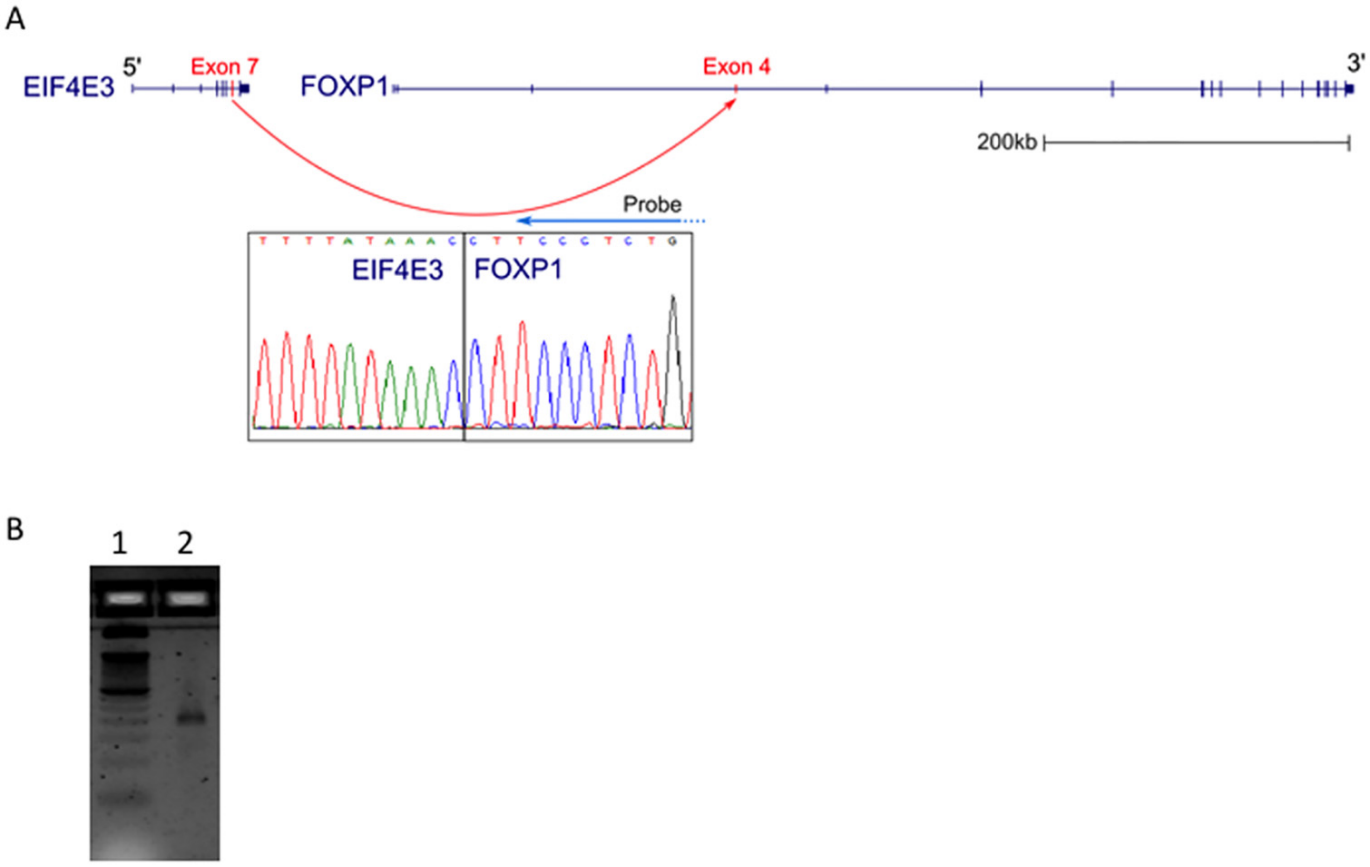
EIF4E3-FOXP1 fusion transcript. (A) Structure of the fusion transcript based on UCSC genome browser tracks. Red arrow indicates the in frame fusion of EIF4E3 exon7 to FOXP1 exon 4. The Sanger sequencing trace below indicates the sequence of the fusion point, while the blue arrow over the sanger sequence trace indicates the 3’ end of the targeting probe. (B) RT-PCR result using PCR primers indicated described in S2 for detecting this fusion transcript. Lane 1: RT-PCR product showing the correct 209bp size, Lane 2: 50bp ladder.

We next sought to test our fusion panel assay on RNA derived from human tissue rather than cultured cells. For this, we utilized human brain reference RNA, a pooled RNA sample derived from 23 healthy donors. Brain tissue has been shown to contain multiple genomic lesions [11] and so we wondered if any of these genomic lesions would lead to the detectable expression of gene fusions. As with UHR RNA, 100 ng of brain reference RNA was used to generate targeted sequencing libraries with Ovation Fusion Panel Target Enrichment System, which were sequenced on an Illumina HiSeq with paired-end, 100base reads. Approximately 7.2M reads were obtained and analyzed for the presence of gene fusions with SOAPFuse. Upon analysis, we identified two potential gene fusions, *ATP5L-MLL* and *EIF4E3-FOXP1*. The *EIF4E3-FOXP1* fusion (Fig 3) again shows the power of only requiring one gene be targeted out of a pair of fused genes as our targeting primers were only designed to *FOXP1*. To our knowledge, this is the first report of *EIF4E3* being fused to *FOXP1* and the fact that this fusion transcript, along with *ATP5L-MLL*, is found in normal tissue suggests that fusion transcripts may occur more often than currently appreciated.

### Gene Fusion Detection from an FFPE RNA Sample

**Fig 4:**
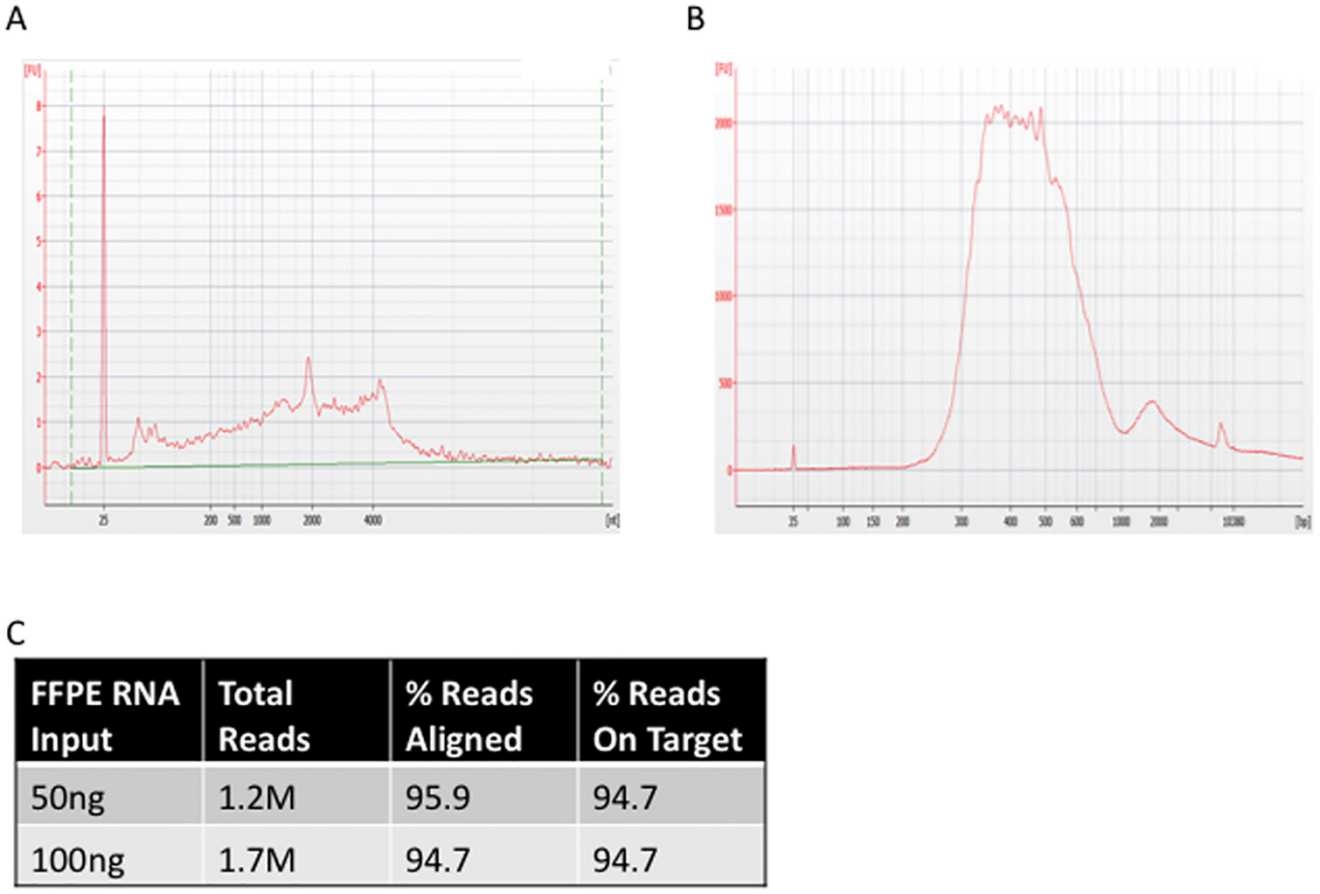
HCC1937 Breast ductal carcinoma RNA. (A) FFPE RNA Bioanalyzer trace. (B) Bioanalyzer trace of sequencing library derived from 100 ng of RNA input shown in A. (C) Sequencing metrics for targeted RNA show that FFPE RNA is efficiently targeted.

Many primary tissue samples are preserved by formalin fixation followed by paraffin embedding (FFPE). This common procedure is known to fragment nucleic acids making it difficult to retrieve sequence data from them. We extracted RNA from HCC1937 primary breast ductal carcinoma cells that had been formalin-fixed and paraffin-embedded (Bioanalyzer trace of RNA in Fig 4A). We used 50 ng and 100 ng of the FFPE RNA in the Ovation Fusion Panel Target Enrichment System assay (resulting library Bioanalyzer trace Fig 4B). Following sequencing 1.2M and 1.6M reads respectively, we were able to identify the known *NFIA-EHF* gene fusion [12] even though we only targeted NFIA and did not have probes targeting EHF in this assay. We again compared our targeted data to a public dataset [13] of 24.6 million untargeted RNA sequencing reads from fresh RNA and found that the untargeted reads identified 11 fusion reads, while the targeted RNA sequencing produced 12 and 20 fusion reads for the 50 ng and 100 ng inputs respectively.

## Discussion

We have applied our customizable SPET technology, previously employed for SNP detection within genomic DNA samples [14], to develop an assay for identification of gene fusion transcripts through targeted RNA sequencing. The Ovation Fusion Panel Target Enrichment System only requires a single primer to hybridize to a fusion transcript in order to identify both gene partners, allowing any potential fusions involving targeted genes to be interrogated in a single tube assay. We have developed a panel to target 401 genes known to form fusion transcripts and have tested the assay on RNA from cell lines, fresh tissue and FFPE samples. In each case we have identified known fusion transcripts and have additionally identified previously unreported fusion transcripts in UHR and normal brain RNA. Furthermore, we show that using a targeted sequencing approach is an efficient mechanism for enhancing the detection of gene fusions compared to standard RNA sequencing suggesting that even samples that have been deeply sequenced by standard RNA sequencing methods would benefit from a targeted approach to identifying gene fusion events.

## Materials and Methods

### Single Primer Enrichment Technology for Gene Fusion Detection

Total RNA was obtained from various sources. RNA derived from the commercial cell line NCI-H2228 (ATCC CRL-5935) was a gift from Susana Ortiz, M.D., Ph.D. (University of California, San Francisco). Universal Human Reference RNA (UHR) (Agilent Technologies) and Human Brain RNA (Ambion) were purchased and used without modification. IRB approval was not required as the human RNA samples used were reference samples obtained from cadavers and purchased from a commercial source. No identifying patient information was obtained by the authors. FFPE sections of cell line HCC1937 were purchased from Alamak Bioscience. 10 micron sections were cut and RNA was extracted from these samples with the Recoverall Total Nucleic Acid Extraction Kit (Ambion). Total RNA was used as input into the Ovation Fusion Panel Target Enrichment System to generate targeted sequencing libraries following the manufacturer’s recommended protocol in the User Guide and described in Fig 1 (PN # 9103 and 9104, NuGEN Technologies, http://www.nugen.com/nugen/index.cfm/products/ovation/ovation-fusion-panel-tes/). Briefly, utilizing the contents of the Ovation Fusion Panel Target Enrichment System kit, 50 or 100 ng of total RNA was reverse transcribed into cDNA. The unpurified cDNA was then made double stranded by the addition of a DNA polymerase. The double stranded cDNA (ds-cDNA) underwent end repair to blunt the ends followed by barcoded adapter ligation. Ligated samples were bead purified and hybridizated with targeting probes starting at 95°C followed by a slow ramp down to 60°C overnight. Following probe annealing, a DNA polymerase was added to the mixture which was heated to 72°C to extend the probes through the target region (see Fig 1). Unextended probes were removed by bead purification and the resulting products were amplified by PCR to create a sequence ready library. Sequencing libraries were quantitated by Bioanalyzer (Agilent Technologies) and Kapa Library Quantification Kit (KAPA Biosytems). Libraries were diluted to 10nM and sequenced on either the Illumina MiSeq or HiSeq platforms as indicated in main text as paired-end reads, either 75 or 100 bases in length (MiSeq and HiSeq respectively).

### Sequence Data Analysis

Resulting sequences were analyzed in the following ways: 1. To determine the reads that were derived from targeted RNA sequences, forward reads were mapped to the Hg19 reference genome using STAR v2.4.0d [15] only allowing unique alignment and allowing chimeric reads. Bedtools intersect [16] was then used to count the number of sequenced reads that were derived from targeted transcripts using default settings, and 2. paired-end sequence data were analyzed for the presence of gene fusions with SOAPFuse v1.22 software [6] with default settings.

For publically available UHR RNA-Seq data, we downloaded SRX333353 [10] and for HCC1937 we downloaded SRX317716 [13].

### Fusion Transcript Validation

Selected putative gene fusions were verified by designing PCR primers around the predicted fusion site. Reverse transcription PCR was used to amplify the predicted fusion gene junctions from the same starting RNA material as was used for sequencing. Resulting PCR products were purified and Sanger sequenced to verify the fusion junction. 200 ng of total RNA input was used in the Ovation cDNA Module For Target Enrichment to generate the cDNA used for the PCR reaction (included as part of the Ovation Fusion Panel Target Enrichment kit). PCR was performed in 50 ul reactions using standard Taq buffer, 0.5mM dNTPs and standard Taq Polymerase (NEB) with 0.4 uM each primer. The PCR reaction was carried out with the following program: 95°C, 3 minutes, followed by 30 cycles of 95°C, 15 seconds, 57°C, 20 seconds and 72°C, 10 seconds. See S2 Table for primer sequences.

### Probe Selection

401 genes were selected for inclusion in the initial gene fusion targeting panel. All refseq exons were input into the proprietary probe selection software with the goal of designing at least two probes targeting each exon. For exons smaller than 300 bases probes were designed so that the 3’ end of the probe would hybridize as near as possible to the known exon boundary, with one probe designed to hybridize to each strand of the double stranded cDNA. For exons larger than 300 bases, additional probes were tiled across the exon at 300 base intervals in order to maximize coverage across these exons. Placing probes near the exon boundaries ensures that extension products will cross at least one exon-exon junction, thus increasing the probability of extending across a gene fusion junction. These design parameters resulted in the generation of 17,999 unique targeting probes across 5769 exons.

## Supporting Information

S1 Table List of Ovation Fusion Panel Target Enrichment System targeted genes. (TXT)

S2 Table PCR primers used for validation of gene fusion events and Sanger sequence of fusions. Forward and reverse primer sequences used in RT-PCR validation. For Sanger sequences, | denotes the breakpoint between sequence aligning to one gene versus the other in the fusion pair.
(TXT)

## References

[1] Forbes S, Beare D, Gunasekaran P, Leung K, Bindal N, Boutselakis H, et al (2014) COSMIC: exploring the world’s knowledge of somatic mutations in human cancer Nucleic Acids Res Advanced Access. cancer.ac.sanger.uk 10.1093/nar/gku1075 PMC4383913 25355519

[2] Rowly JD (1973) Letter: A new consistent chromosomal abnormality in chronic myelogenous leukaemia identified by quinacrine fluorescence and Giemsa staining. Nature 243(5405):290–3. 4126434 10.1038/243290a0

[3] Heisterkamp N, Stam K, Groffen J, De Klein A, Grosveld G (1985) Structural organization of the *bcr* gene and its role In the Ph’ translocation. Nature 315:758–761. 2989703 10.1038/315758a0

[4] Liehr T (2010) Fluorescence In Situ Hybridization (FISH)–Quality Issues In Cytogenetics; p. 315–320. In Kristoffersson U., Schmidtke J. and Cassiman J. (Eds.), Quality Issues In Clinical Genetic Services, Springer Netherlands, Houten, Netherlands.

[5] Ozsolak F, Milos PM (2011) RNA sequencing: advances, challenges and opportunities. Nat Rev Genet 12:87–98. 10.1038/nrg2934 21191423 PMC3031867

[6] Jia W, Qui K, He M, Song P, Zhou Q, Zhou F, et al (2013) SOAPFuse: an algorithm for identifying fusion transcripts from paired-end RNA-seq data. Genome Biology 14:R12 10.1186/gb-2013-14-2-r12 23409703 PMC4054009

[7] Kim P, Yoon S, Kim N, Lee S, Ko M, Lee H, et al (2009) ChimerDB 2.0 –a knowledgebase of fusion genes updated. Nucleic Acids Res. 38:D81–D85. 10.1093/nar/gkp982 19906715 PMC2808913

[8] Kim D, Salzberg S (2011) TopHat-Fusion: an algorithm for discovery of novel fusion transcripts. Genome Biology 12:R72 10.1186/gb-2011-12-8-r72 21835007 PMC3245612

[9] Chinnaiyan A, Kumar-Sinha C, Robinson D, Kalyana-Sundaram S (2011)Recurrent gene fusions in breast cancer. US patent US20130096021.

[10] Rapaport F, Khanin R, Liang Y, Pirun M, Krek A, Zumbo P, et al (2013) Comprehensive evaluation of differential gene expression analysis methods for RNA-seq data. Genome Research 14:R95. 10.1186/gb-2013-14-9-r95 PMC4054597 24020486

[11] McConnell MJ, Lindberg MR, Brennand KJ, Piper JC, Voet T, Cowing-Zitron C, et al (2013) Mosaic copy number variation in human neurons. Science 342:632–637. 10.1126/science.1243472 24179226 PMC3975283

[12] Ha KCH, Lalonde E, Li L., Cavallone L, Natrajan R, Lambros MB, et al (2011) Identification of gene fusion transcripts by transcriptome sequencing in *BRCA1*- mutated breast cancers and cell lines. BMC Med Genomics 4:75 10.1186/1755-8794-4-75 22032724 PMC3227591

[13] Daemen A, Griffith OL, Heiser LM, Wang NJ, Enache OM, Sanborn Z, et al (2013) Modeling precision treatment of breast cancer. *Genome Biology* 14(10):R110. 24176112 10.1186/gb-2013-14-10-r110 PMC3937590

[14] Durinck S, Stawiski E, Pavia-Jimenez A, Modrusan Z, Kapur P, Jaiswal BS, et al (2014) Spectrum of diverse genomic alterations define non-clear cell renal carcinoma subtypes. Nature Genetics. 10.1038/ng.3146 PMC4489427 25401301

[15] Dobin A, Davis CA, Schlesinger F, Drenkow J, Zaleski C, Jha S, et al (2012) STAR: ultrafast universal RNA-seq aligner. Bioinformatics 29:15–21. 10.1093/bioinformatics/bts635 23104886 PMC3530905

[16] Quinlan AR, Hall IM (2010) BEDTools: a flexible suite of utilities for comparing genomic features. Bioinformatics 26:841–842. 10.1093/bioinformatics/btq033 20110278 PMC2832824

